# Key Factors Related to Short Course 100 m Breaststroke Performance

**DOI:** 10.3390/ijerph17176257

**Published:** 2020-08-27

**Authors:** Bjørn Harald Olstad, Henrik Wathne, Tomohiro Gonjo

**Affiliations:** Department of Physical Performance, Norwegian School of Sport Sciences, 0863 Oslo, Norway; hwathne54@gmail.com (H.W.); tomohirog@nih.no (T.G.)

**Keywords:** swimming race analysis, automatic, kinematics, segments, techniques

## Abstract

Background and aim: To identify kinematic variables related to short course 100 m breaststroke performance. Methods: An automatic race analysis system was utilized to obtain start (0–15 m), turn (5 m before the wall until 10 m out), finish (95–100 m), and clean swimming (the rest of the race) segment times as well as cycle rate and cycle length during each swimming cycle from 15 male swimmers during a 100 m breaststroke race. A bivariate correlation and a partial correlation were employed to assess the relationship between each variable and swimming time. Results: Turns were the largest time contributor to the finishing time (44.30 ± 0.58%), followed by clean swimming (38.93 ± 0.50%), start (11.39 ± 0.22%), and finish (5.36 ± 0.18%). The finishing time was correlated (*p* < 0.001) with start segment time (r = 0.979), clean swimming time (r = 0.940), and 10 m turn-out time (r = 0.829). The clean swimming time was associated with the finishing time, but cycle rate and cycle length were not. In both start and turns, the peak velocity (i.e., take-off and push-off velocity) and the transition velocity were related to the segment time (r ≤ −0.673, *p* ≤ 0.006). Conclusions: Breaststroke training should focus on: (I) 15 m start with generating high take-off velocity, (II) improving clean swimming velocity by finding an optimal balance between cycle length and rate, (III) 10 m turn-out with maintaining a strong wall push-off, and (IV) establishing a high transition velocity from underwater to surface swimming.

## 1. Introduction

In competitive swimming, the goal is to finish the race in the least possible time. Race analysis, therefore, provides an objective and quantifiable breakdown of a race into smaller segments for a detailed analysis of the performance [1]. This analysis also provides the most comprehensive evaluation of a swimmer’s true state of readiness [2]. The race is often broken down into four main segments: the start, the clean swimming, the turn(s) and the finish [2,3]. The start contributes more to the finishing time in sprint events, compared with longer distance races [4], and the highest velocity occurs during this segment. The turn(s) contributes more to the finishing time in short course than long course pools. This is also where the second highest velocity during a race is achieved due to the wall push-off, and the segment performance is affected by the race distance [5].

Previous research has identified factors contributing to the time spent in these segments, e.g., start (block position, reaction/block time, push-off, flight, entry and underwater) [6,7,8,9], turn (5 m approach, pivot time, push-off, underwater distance and velocity) [10,11,12], clean swimming velocity (cycle length and cycle rate) [13,14], and the finish (timing of the wall touch and the trajectory of the hands) [15]. Therefore, the winner of a race or the swimmer with the fastest finishing time is not always the swimmer with the highest mean clean swimming velocity, but rather the swimmer who performs well on all of the race segments [2].

Despite a large amount of literature on swimming race analysis, knowledge on this topic should constantly be updated due to frequent changes in competition rules, such as the restriction of underwater swimming distance and swimsuit as well as the introduction of new equipment for starts. One recent and major change in swimming competition rules includes the execution of one dolphin kick at any time prior to the first breaststroke kick after the start and turns, which became effective in December 2014.

Segments affecting the finishing time have been investigated in the men’s 100 m long course breaststroke [3,5,12,16,17]. Two studies investigated the contribution of different segments to the finishing time in the men’s short course breaststroke [18,19]. In short course, approximately 62% of the finishing time in the 100 m breaststroke came from start, turn, and finish segments [18], while the turns compiled 39% of the total finishing time in the 200 m breaststroke [19]. However, there has been no short course breaststroke race analysis conducted after the bespoken rule change relating to breaststroke start and turns. Given the high contribution of short course start and turn segments, the effect of each race segment on short course performance with current competition rules should be analyzed.

Most race analysis is either tracked in specialized software or computed from digitizing video footage from multiple cameras with a manual intervention combined with official split and race times from an electronic timing system [3,5,10,18,20,21]. Such approaches allow coaches and researchers to investigate the contribution of race segments to the total race performance with either fixed-distance-based (start segment, 0–15 m; turn segment, 15 m around the wall; finish segment, last 5 m of the race; clean swimming segment, the rest of the race) or individual-distance-based (start segment, start to the breakout point; turn segment, from the beginning of the turn motion until the following breakout; clean swimming segment, the rest of the race) segment definitions [22,23]. However, the contribution of segments to the total race time should be interpreted carefully, since each segment is not independent but related to each other [22]. For instance, a large clean swimming velocity might be affected by the large initial velocity obtained by start and turns; in such a case, a simple contribution calculation based on segment times might cause a slight overestimation of the importance of the clean swimming segment. A partial correlation analysis potentially minimizes this concern. Unlike the bivariate correlation, partial correlation measures the degree of association between two variables with removing the effect of a set of controlling variables. In the clean swimming example provided above, the use of partial correlation analysis would provide how much the clean swimming segments are related to the total swimming time without the effect of start and turn segments. 

Therefore, the purpose of the study was to identify segmental factors related to the finishing time in the modern 100 m short course breaststroke. Assuming that the recent rule change in breaststroke start and turn positively affects the swimming performance, it was hypothesized that start and turn segments would account for less time in relation to the total time compared with a previous study [18] conducted before the rule change. Similarly, from the presumptive perspective that higher-level swimmers would adapt themselves to the rule change better than lower-level swimmers, it was also hypothesized that start and turn segments would show a large partial correlation with the total swimming time.

## 2. Materials and Methods

### 2.1. Participants

Fifteen high-level male breaststroke swimmers (mean ± SD age 19.0 ± 2.5 years; height 183.9 ± 5.9 cm; body mass 79.8 ± 7.8 kg; personal record short course 63.20 ± 2.69 s; Fédération Internationale de Natation (FINA) points 688.1 ± 86.8 points) participated voluntarily. Five were in the final of the seasonal championship the day before the experiment. All of them were familiar with the testing procedures and wore a yellow swim cap and a non-bright color swimsuit for automatic head displacement recognition. This study was approved by the local ethical committee of the Norwegian School of Sport Sciences (approved in 2018 with approval number 46–060218–200318) and the National Data Protection Agency for Research (approved in 2018 with approval number 58650) and was conducted in accordance with the standards set by the Declaration of Helsinki. All participants provided an informed consent before data collection.

### 2.2. Experimental Testing Design

The experiment took place in a 25 m indoor swimming pool (2–4 m deep) with constant environmental conditions (water temperature 27 °C, air temperature 29 °C, and humidity 55%). Before the experiment, all participants performed their personal warm-up procedure as they do before a competition on land and in the water. All participants swam 100 m breaststroke with maximal effort with the goal of finishing in the fastest possible time.

### 2.3. Data Collection

**Figure 1:**
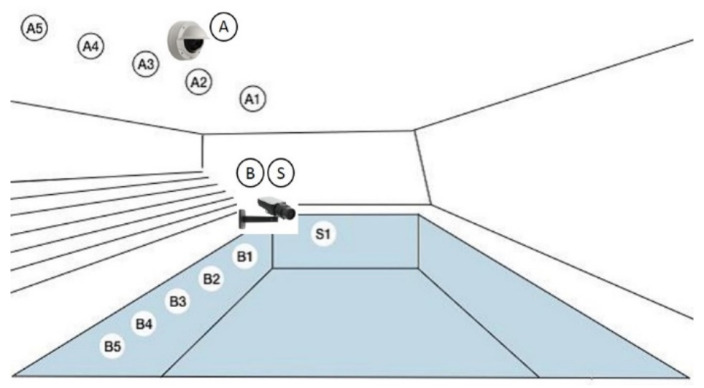
Placement of the video cameras. Note: A—The above water cameras (locations A1–A5), B and S—the underwater cameras, placed behind windows (locations B1–B5 and S1 placed beneath the starting block).

Breaststroke kinematics (Table 1) were analyzed using a race analysis system with automatic movement recognition and data processing (AIMsys Sweden AB, Lund, Sweden). The system consisted of 11 stationary digital video cameras. Five cameras were mounted above water, and five were mounted beneath the water surface (behind windows) along with the 25 m pool with 5 m apart. The first and the fifth camera both above and underwater were placed 2.5 m away from the end walls of the pool. The last camera was beneath the water surface (behind a window) in the middle of the line at the starting side (Figure 1). All underwater cameras were placed perpendicular to the swimming direction 0.70 m beneath the water surface. The distance from the underwater cameras on the side to the race lane was 5.5 m. The above water cameras were mounted on the wall approximately 5 m over the water surface facing down. The cameras were Axis Q3505-VE Mk II Network Camera (Axis AB, Lund, Sweden) above water and Axis Q1635 Network Camera (Axis AB, Lund, Sweden) underwater (Figure 1). The sampling frequency was 50 Hz, and the camera resolution was 1080 p. Performance times (1/100 s) came from an electronic timing system (Omega, Bienne, Switzerland) integrated with the camera system.

#### Synchronization

Synchronization of the cameras is done in the analysis system based on the cameras’ internal clocks being precisely synchronized. This is an automatic process that occurs when the cameras are turned on. The race recording will not start if the system server cannot connect to the Omega timing system or if any of the cameras or camera clocks are found to be out of synchronization.

### 2.4. Calibration

**Figure 2:**
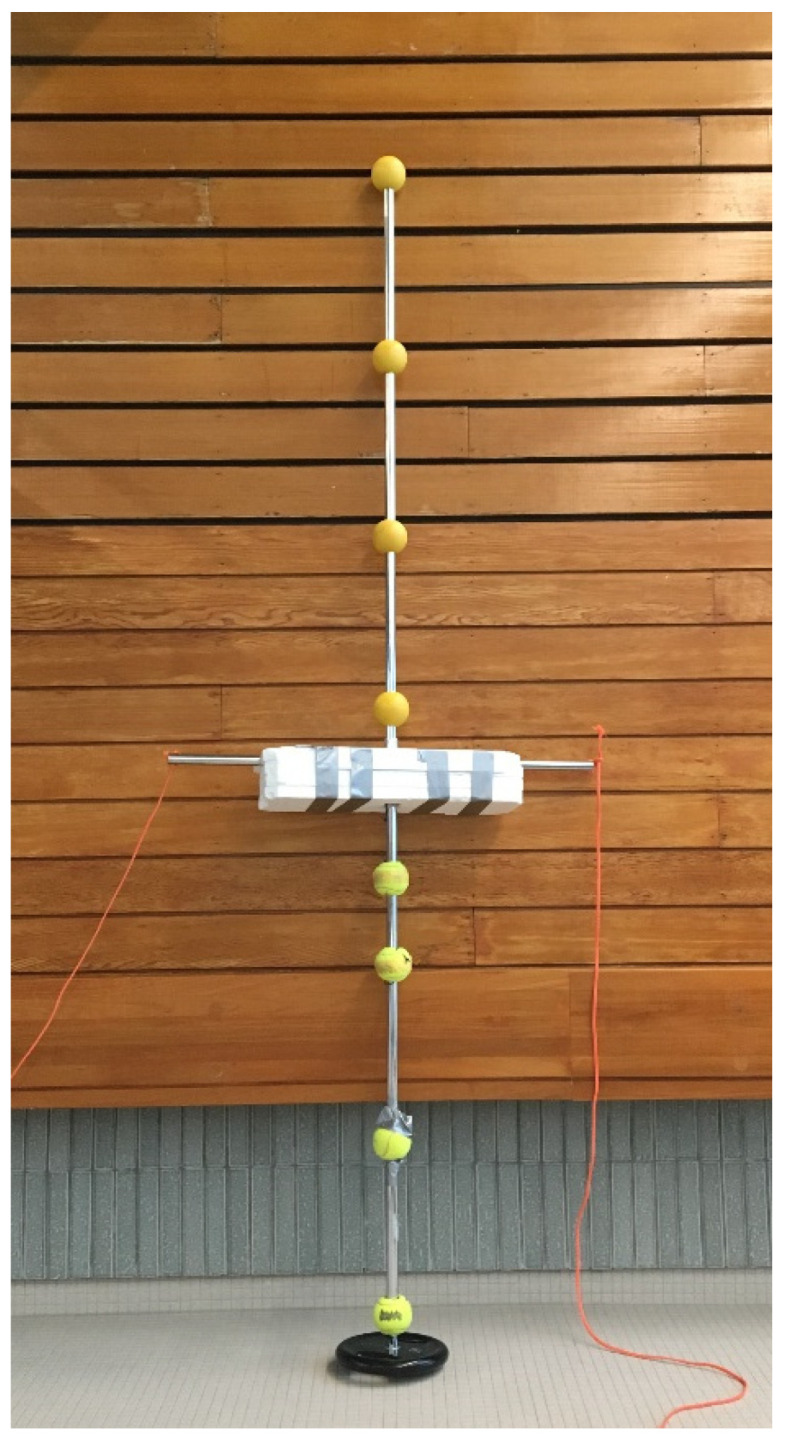
The semi-submersed rigid calibration object consisting of eight bright-yellow balls with fixed distances separated by a polystyrene foam flotation device. Two ropes of polyester were attached to each side at the water level for pulling the calibration object through the swimming pool.

Calibration was conducted with a custom-built semi-submersed rigid calibration object consisting of eight bright-yellow balls (four mounted above and under the water surface; Figure 2). The distances (cm) between the center of the above-water balls were: 47.0, 44.5, and 41.5 from the top and 10.0 between the center of the last ball and the top of the polystyrene cube. The thickness of the polystyrene foam flotation device cube was 11.5 cm. The distances (cm) between the underwater balls were: 17.0 (between the bottom of the cube and the center of the first ball) and thereafter 19.0, 38.0, and 36.0 from the top. This ensured that different parts of the object were simultaneously viewed by the above and underwater camera. Two ropes of polyester were attached to each side at the water level for pulling the calibration object through the swimming pool. The object was pulled in a zigzag pattern starting at the wall in the middle of the racing lane and thereafter with an approximate 1–2 m of horizontal displacement from left to right covering the 25 m pool length. The calibration process consisted of the following five steps and was performed by the manufacturer: (1) intrinsic in-air calibration of all cameras, (2) capture calibration object, (3) detect calibration markers, (4) initialize calibration object pose and camera extrinsic parameters, and (5) refine parameters using bundle adjustment (described in detail by a previous study [24]). The mean reprojection error for the calibration object was 2.0 pixels, corresponding to a 0.025° angular error and 0.006 m linear error.

### 2.5. Statistical Analyses

Normality of the distribution was checked for all variables using the Shapiro–Wilk test before further analysis. The condition of normal distribution was not met for transition cycle length during start, glide distance for turns total and number of swimming cycles and cycle length on lap 1, stroke cycles per lap, and velocity 5–10 m on lap one and three. A descriptive analysis (mean and standard deviation) was conducted for all race factors. The different segments and underlying factors were time normalized to finishing time and lap time.

The Pearson’s bivariate and partial correlation coefficients (r) were used to indicate relationships between variables in two ways: (1) between the time of each segment with the criterion variable total time and (2) between underlying performance factors within a segment with the criterion variable segment time. Spearman’s rho rank correlation was used for nonparametric data. The threshold values of the correlation coefficient were 0.0–0.1 for trivial, 0.1–0.3 for small, 0.3–0.5 for moderate, 0.5–0.7 for large, 0.7–0.9 for very large, and 0.9–1.0 nearly perfect, according to recommendations in the literature [25]. Significance levels were considered as *p* < 0.05. All statistical analyses were conducted using IBM SPSS Statistics version 24 (IBM Corp, Armonk, NY, USA).

## 3. Results

Results from the correlation analyses conducted in the present study are shown in Table 2, Table 3, Table 4, Table 5, Table 6, Table 7 and Table 8. Investigation related to the finishing time of the race exhibited a nearly perfect correlation for 15 m start time and clean swimming time with the total finishing time (r = 0.979 and 0.940, respectively, *p* < 0.001). The 10 m turn-out time also showed a very large correlation with finishing time (r = 0.829, *p* < 0.001). On the other hand, 5 m turn-in and 95–100 m finish time did not have an association with the total finishing time (Table 2). 

In the start segment, there were very large negative correlations of the flight distance and the transition velocity with the segment time (r = −0.804 and −0.733, *p* ≤ 0.002). A large negative correlation coefficient with this segment time was also observed in the breakout distance and the peak velocity (r = −0.652 and −0.673, *p* = 0.008 and 0.006, respectively) (Table 3). In the clean swimming segment (Table 4), mean cycle velocity had a very large or nearly perfect correlation with the segment time in all laps (r ≤ −0.885, *p* < 0.001). On the other hand, mean cycle length and rate did not have a significant relationship with the segment time. During the 5 m turn-in segment, no analyzed variables exhibited a significant correlation with the segment time (Table 5). On the other hand, in the 10 m turn-out segment (Table 6), the peak velocity and the transition velocity had a significant correlation with the segment time in all laps (r and *p* value ranged from −0.755 to −0.535 and from 0.001 to 0.040, respectively). The time for the 10 m turn-out increased throughout the race. A mean increase of 0.38 ± 0.09 s occurred between the first and the last turn, while pivot time increased by 0.04 ± 0.06 s. Breakout time and distance were reduced by 0.08 ± 0.32 s and 0.54 ± 0.38 m, respectively. During the finish segment (Table 7), no analyzed kinematic variables had a significant relationship with the segment time. In Table 8, start segment velocity showed very large negative correlations of 0–5 m (r = −0.879, *p* < 0.001), 5–10 m (r = −0.745, r = 0.002), and 10–15 m (r = −0.776, *p* = 0.001) with the finishing time. The last 5 m of each lap (5 m turn-in and 95–100 m velocity), only the first lap showed a significant relationship with the finishing time, whereas other laps did not exhibit significant relationships between these variables. Swimmers showed a significant correlation of the 5 m segment velocity with the finishing time in most of the 5 m segments (r and *p* value ranged from −0.978 to −0.651 and from <0.001 to 0.009, respectively) apart from 0–5 m in the fourth lap and 20–25 m in the second, the third, and the fourth laps. 

Partial contribution from the different segments to the finishing time is presented in Table 9. The turn segment (5 m turn-in, 10 m turn-out, and pivot time) was the largest time contributor to the finishing time (44.30 ± 0.58%), and the breakout time from the start and turns contributed to the finishing time by 35.85 ± 3.98%. Clean swimming, 15 m start, and 5 m finishing time had contributions to the finishing time by 38.93 ± 0.50%, 11.39 ± 0.22%, and 5.36 ± 0.18%, respectively.

## 4. Discussion

The purpose of the current study was to identify segmental factors related to the 100 m short course breaststroke race time. Two hypotheses were initially set in the current study; non-swimming segments would account for less time relative to the total time compared with Kjendlie et al. [18], and start and turn segments would show a correlation with the total swimming time. The contribution of the start segment in the present study was about 1% smaller than that reported in Kjendlie et al. [18], while the contribution of turns was very similar. Thus, the first hypothesis of the present study was partly accepted. Nearly perfect or very large correlations with the 100 m finish time observed in the 15 m start time and the 10 m turn-out time suggest that fast swimmers have better start and 10 m turn-out skills than slower swimmers, which supports our second hypothesis. Even though the 15 m start does not compile most of the finishing time, it appeared to have the strongest relationship with the final performance. 

The starting segment has a large impact on the finishing time and the swimming velocity on the first lap regardless of stroke, especially for short race distances such as 50 and 100 m [4]. It has been reported that, in long course, the 15 m start segment has a large effect on the finishing time (r = 0.870), and better swimmers generally have a better start technique than less skilled swimmers [5]. In the present study, the finishing time was correlated with the first 0–5 m, 5–10 m, and 10–15 m mean velocity with r values of −0.879, −0.745, and −0.776, respectively. The slight change in the correlation coefficient from the 0–5 m to the next two 5 m segments might suggests the importance of the beginning of the start segment. This was also supported by a large and very large negative correlation of the peak velocity during the start and the flight distance with the 15 m time. 

The importance of the flight distance was consistent with Peterson Silveira et al. [8], who reported a strong relationship (r ≈ −0.895) between the flight distance in front and rear kick starts and the 5 m time. The peak velocity investigated in the present study was the peak head velocity and not the center of mass velocity. Thus, it was not only affected by the impulse swimmers obtained from the starting block but also the head velocity relative to the center of mass. In fact, the peak velocity during the start segment in the present study (4.71 ± 0.25 m/s) was about 0.3–0.6 m/s faster than the take-off velocity reported in previous studies [26,27], which suggests the potential effect of the head motion relative to the center of mass due to joint motions such as the hip extension. However, given that the contribution of the trunk angular momentum to the whole-body angular momentum is only about 7% during the start [27], it is likely that the effect of assessing the head instead of the center of mass is small. Furthermore, considering that swimmers generally produce hip extension torque and rarely produce hip flexion torque throughout the on-block motion [28], the error due to the head velocity analysis should be systematic. In other words, even though the absolute peak velocity in the present study does not represent the take-off velocity, the correlation with the 15 m time should be comparable with the potential relationship between the take-off velocity and the 15 m time, i.e., the large correlation of the peak velocity and the 15 m time in the present study implies the importance of the take-off velocity. This is reasonable since the literature also suggests the take-off velocity as the most important factor to predict the 15 m time [29].

Breakout distance also had a large negative correlation with the 15 m time, whereas the breakout time did not exhibit a significant association with the 15 m time. These results agree with a previous study that investigated start and turn performance in international long course 100 m breaststroke races [12], where significant negative correlation (−0.17 for male and −0.27 for female) of the breakout distance and the finishing time are reported. However, the correlation coefficient of the present study (−0.652) was much larger than the previous study. This difference might be due to the range of the investigated athletes’ level. In the present study, the FINA points of the participants ranged from 562 –811, which means that the level of the swimmers ranged from regional to international levels. On the other hand, the swimmers tested in the previous study [12] were all finalists or semi-finalists of the FINA 2013 World Swimming Championships. It is evident that the range of swimmers’ levels was much wider in the current study compared with the previous study, and it is possible that the difference affected the magnitude of the correlation coefficient. 

The present study found that the 5–10 m and the 10–15 m mean velocities are important determinants for the finishing time, even with controlling the effect of the preceding segment velocity. These outcomes imply the importance of the underwater phase in the start segment. From the correlation analysis, it was evident that the transition stroke was an important factor for the start segment (0–15 m). However, considering that the breakout distance was around 13 m in the present study, the transition stroke could only explain why 10–15 m segment was related to the finishing time. Therefore, it is not entirely clear what the determinants of the underwater phase performance are. After the entry until the breakout, swimmers perform the glide, the pull-out motion that includes the backward pull motion of the arm as well as a dolphin kick, and the arm and leg recovery motion [30]. Further studies are required to assess which of those factors affect the start segment performance. 

Among the main segments, turns (5 m turn-in, pivot time, and 10 m turn-out) showed the highest relative time contribution to the finishing time with 44.30%. A previous study among finalists in the European long course championship identified the turn as an important contributor but a less substantial factor to the finishing time with 19.96% [3]. This is largely because long course only permits one turn opposed to three in short course. Within the turn segment, total 10 m turn-out exhibited a very large correlation with the finishing time, whereas no relationship was found between total 5 m turn-in and finishing time. The 10 m turn-out summed 25.43% of the finishing time and nearly equaled the breakout distance (9.18 m). In this part of the segment, the second highest velocity of the race occurred during the first underwater streamline (2.96 m/s). This peak velocity and the mean cycle velocity of the transition stroke had large to very large correlations with the 10 m turn-out. Similar to the peak velocity in the start segment, the peak velocity in the turn segment is also not the center of mass velocity but the head velocity. However, given that the push-off is the fastest moment in the turn segment that is performed with a streamline posture that includes minimal limb motion [31], the head peak velocity should be very similar to the center of mass velocity at the push-off from the wall. It is therefore important for swimmers to take advantage of this velocity by executing the best possible streamlined position to minimize drag during the first glide after push-off [32,33]. Nevertheless, the same limitation as in the start segment should also be noted for the turn segment. The present study cannot give insight into detailed kinematic factors during the underwater motion in the 10 m turn-out, which should be further investigated. 

In the present study, swimmers exhibited an increase in 10 m turn-out time by 0.38 s from the first to the last lap. Given that the pivot time raised by only 0.04 s, the decrease in the turn-out performance was primarily due to after the pivot motion. In the turn-out phase, breakout time and distance were reduced by 0.08 ± 0.32 s and 0.54 ± 0.38 m, respectively, meaning that it resulted in a reduction of the mean velocity until the breakout by approximately 0.06–0.07 m/s. This can be explained by a weaker push-off that is evident from the drop of the peak velocity (about 0.1 m/s) between the first and the last turn. This also supports the velocity decrease between 0–5 m and 5–10 m in the last turn, which was also about 0.1 m/s less than the first turn. Those results imply the effect of fatigue.

Clean swimming showed the second highest relative time contribution to the finishing time with a very large correlation with the finishing time. Clean swimming velocity is the product of cycle length and cycle rate [34], and cycle length is often defined as the most important factor for increasing swimming velocity [35,36]. Interestingly, however, there was no significant correlation observed between cycle length and clean swimming time nor between cycle rate and clean swimming time, which indicates that fast swimmers do not necessarily exhibit long cycle length, but each swimmer has their own strategies to achieve high clean swimming velocity. 

Only the first lap showed a significant relationship of the last 5 m velocity with the finishing time, and the other laps did not exhibit such relationships. Neither the number of cycles in the 5 m range nor the glide distance before the wall touch showed a significant correlation with the last 5 m time, whereas the mean cycle velocity did not have a significant relationship with the 5 m time in all laps. These results cannot explain why only the last 5 m velocity in the first lap had a very large correlation with the finishing time, and it is difficult to conclude the source of the difference between the first lap and the remaining laps. One possible explanation might be related to a race strategy of the swimmers. The results showed a larger clean swimming velocity between the 15–20 m in the first lap compared with other 5 m ranges in the clean swimming segment (10–15 m and 15–20 m in the second, the third, and the fourth laps). Furthermore, the 15–20 m velocity in the first lap was much faster than the precedent 5 m where the breakout was performed, whereas this was not the case in the other laps. These results might suggest that swimmers primarily focused on producing a high velocity after the breakout in the first lap throughout the lap. In other words, perhaps they were focusing on maximizing the velocity rather than “adjusting” their motion for wall touch, but in the other laps, they might have focused on efficient wall touch rather than maximizing the velocity. Nevertheless, there is little evidence available for swimming finish segment [22], and the described possibility is merely a conjecture. Future studies should investigate how swimmers adjust their stroke kinematics for a good turn segment performance. 

## 5. Conclusions

The specific findings from this study contribute to a better understanding of which factors are related to performance and how much each segment contributes to the 100 m short course breaststroke finishing time. Based on the results of this study, we suggest that coaches and swimmers should focus on (I) the 15 m start, (II) finding an optimal balance between cycle length and rate to achieve a large clean swimming velocity, rather than focusing on improving cycle length, and (III) the overall turns with a focus on the 10 m out with a strong wall push-off.

## Figures and Tables

**Table 1:** Definition of kinematical variables chosen for analysis.

Segments and Parameters	Calculation
100 m performance time (s)	The total finishing time
Segment contribution (%)	The time spent in the respective segments in relation to the 100 m performance time
5 m segment velocity (m/s)	Mean forward head velocity for each 5 m segment throughout the 100 m race
*Start segment*	
15 m time (s)	Time taken from the starting signal until the head reaches the 15 m mark
Block time (s)	Time taken from the starting signal until the toes leave the starting block
Flight time and distance (s, m)	Time and distance from the toes leaving the starting block until the hands enter the water
Breakout time and distance (s, m)	Time taken from the starting signal until the head breaks the water surface.
Peak velocity (m/s)	The highest velocity during the start segment
Transition velocity (m/s)	Mean swimming velocity during the transition cycle from the underwater to the surface swimming
Transition cycle length (m/cycle)	Cycle length during the transition cycle from the underwater to the surface swimming
Transition cycle rate (cycles/min)	Cycle rate during the transition cycle from the underwater to the surface swimming
*Turn segment (turn-in)*	
5 m turn-in time (s)	Time taken from the head reaches the last 5 m mark until the wall-touch
Mean cycle velocity (m/s)	Mean swimming velocity during complete swimming cycles in the 5 m segment
Mean cycle length (m/cycle)	Mean cycle length during complete swimming cycles in the 5 m segment
Mean cycle rate (cycles/min)	Mean cycle rate during complete swimming cycles in the 5 m segment
Number of cycles	The number of swimming cycles during the 5 m segment
Glide distance (m)	The distance from the wall to the head at the end of the last swimming cycle in the lap
*Turn segment (turn-out)*	
10 m turn-out time (s)	Time taken from the wall-touch until the head reaches the 10 m mark
Pivot time (s)	From the hands touch the wall until the feet touch the wall (lap 1–3)
Water breakout time and distance (s, m)	Time and distance from the feet pushing-off the wall until the head breaks the surface (lap 2–4).
Peak velocity (m/s)	The highest velocity observed between from the wall-touch and the head breaking the water surface (lap 2–4)
Transition velocity (m)	Mean swimming velocity during the transition cycle from the underwater to the surface swimming
Transition cycle length (m/cycle)	Cycle length during the transition cycle from the underwater to the surface swimming
Transition cycle rate (m)	Cycle rate during the transition cycle from the underwater to the surface swimming
*Start + turn segment*	
15-m time + total turns (s)	From the starting signal until the head reaches the 15-m mark + From the head reaching the 5 m mark before the wall (lap 1–3) to 10 m after the wall (lap 2–4)
*Swim segment*	
Clean swimming time (s)	Time taken during the segment (15–25 m for lap 1 and from 10–20 m for laps 2–4)
Clean cycle velocity (m/s)	Mean velocity during complete cycles during the segment (15–25 m for lap 1 and from 10–20 m for laps 2–4)
Mean cycle length (m/cycle)	Mean cycle length during complete cycles during the segment
Mean cycle rate (cycles/min)	Mean cycle rate during complete cycles during the segment
Number of strokes	Number of cycles during the segment
*Finish segment*	
5-m finish time (s)	Time taken from the head reaching the 95-m mark until the hands touch the wall
95-m velocity (m/s)	Velocity measured when the head reaches the 95-m mark
Clean cycle velocity (m/s)	Mean velocity during complete cycles during the segment
Mean cycle length (m/cycle)	Mean cycle length during complete cycles during the segment
Mean cycle rate (cycles/min)	Mean cycle rate during complete cycles during the segment
Number of cycles	Number of cycles during the last 5 m of the race
Glide distance (m)	The distance from the wall to the head at the end of the last swimming cycle of the race
*Position data*	Detector uncertainty is estimated below 1 cm
Head position	The head detector has been trained to predict the image projection of the center of the head

**Table 2:** Descriptive statistics (mean ± standard deviation) and partial correlation analysis of final performance time, start, turn, swim and finish segments with underlying components.

Segments and Factors	Total	r; *p*-Value	Lap 1	r; *p*-Value	Lap 2	r; *p*-Value	Lap 3	r; *p*-Value	Lap 4	r; *p*-Value
100 m finish time (s)	64.84 ± 3.17	n.a.	13.78 ± 0.80	**0.968**; **<0.001**	16.46 ± 0.83	**0.845**; **<0.001**	16.98 ± 0.82	**0.722**; **0.004**	17.62 ± 0.80	**0.666**; **0.009**
15 m start time (s)	7.39 ± 0.47	**0.979**; **<0.001**	7.39 ± 0.47	**0.980**; **<0.001**	n.a.	n.a.	n.a.	n.a.	n.a.	n.a.
Clean swimming time (s)	25.24 ± 1.18	**0.940**; **<0.001**	3.36 ± 0.16	**0.905**; **0.001**	7.04 ± 0.36	**0.975**; **<0.001**	7.28 ± 0.35	**0.973**; **<0.001**	7.57 ± 0.35	**0.992**; **<0.001**
5 m turn-in (s)	9.60 ± 0.62	0.280; 0.333	3.03 ± 0.20	**0.747**; **0.002**	3.23 ± 0.24	0.533; 0.050	3.34 ± 0.22	0.261; 0.368	n.a.	n.a.
10 m turn-out (s)	19.13 ± 0.95	**0.829**; **<0.001**	n.a.	n.a.	6.19 ± 0.31	**0.865**; **<0.001**	6.36 ± 0.36	**0.850**; **<0.001**	6.57 ± 0.30	**0.832**; **<0.001**
95–100 m finish time (s)	3.47 ± 0.23	−0.307; 0.286	n.a.	n.a.	n.a.	n.a.	n.a.	n.a.	3.47 ± 0.23	−0.274; 0.344

*Note:* n.a.—it does not apply. *p* < 0.05 together with its corresponding r is **bolded**. Pearson correlation coefficients for 15 m start and 10 m turn-out time with the 100 m finish and lap time. Remaining variables are partial correlation coefficients with the time of the previous segment as control variable.

**Table 3:** Descriptive statistics (mean ± standard deviation) and correlation analysis of the start segment with underlying components.

Segments and Factors	Total	r; *p*-Value
*15 m start time (s)*	7.39 ± 0.47	n.a.
Block time (s)	0.68 ± 0.03	0.474; 0.074
Flight time (s)	0.38 ± 0.05	−0.354; 0.196
Flight distance (m)	3.18 ± 0.21	**−0.804; <0.001**
Breakout time (s)	6.02 ± 0.51	−0.090; 0.749
Breakout distance (m)	13.06 ± 1.02	**−0.652**; **0.008**
Peak velocity (m/s)	4.71 ± 0.25	**−0.673**; **0.006**
Transition velocity (m/s)	1.41 ± 0.08	**−0.733**; **0.002**
Transition cycle length (m/cycle)	1.63 ± 0.12	−0.404; 0.136
Transition cycle rate (cycles/min)	52.29 ± 3.88	−0.236; 0.398

*Note:* n.a.—it does not apply. *p* < 0.05 together with its corresponding r is **bolded**. Pearson correlation coefficients with 15 m start time. Spearman’s rho rank correlation coefficient for transition cycle length with 15 m start time.

**Table 4:** Descriptive statistics (mean ± standard deviation) and partial correlation analysis of the clean swimming segment time with underlying components.

Segments and Factors	Total	r; *p*-Value	Lap 1	r; *p*-Value	Lap 2	r; *p*-Value	Lap 3	r; *p*-Value	Lap 4	r; *p*-Value
*Clean swimming time (s)*	25.24 ± 1.18	n.a.	3.36 ± 0.16	n.a.	7.04 ± 0.36	n.a.	7.28 ± 0.35	n.a.	7.57 ± 0.35	n.a.
Mean cycle velocity (m/s)	1.41 ± 0.06	**−0.966**; **<0.001**	1.51 ± 0.07	**−0.885**; **<0.001**	1.43 ± 0.07	**−0.919**; **<0.001**	1.38 ± 0.07	**−0.976**; **<0.001**	1.32 ± 0.06	**−0.968**; **<0.001**
Mean cycle length (m/cycle)	1.67 ± 0.11	−0.174; 0.535	1.71 ± 0.11	0.107; 0.704	1.71 ± 0.13	−0.094; 0.740	1.68 ± 0.12	−0.189; 0.499	1.58 ± 0.13	−0.138; 0.625
Mean cycle rate (cycles/min)	50.89 ± 3.87	−0.392; 0.149	53.28 ± 4.01	−0.472; 0.076	50.33 ± 4.45	−0.426; 0.113	49.62 ± 4.04	−0.401; 0.138	50.32 ± 4.40	−0.322; 0.241
Number of cycles	25.21 ± 1.63	0.247; 0.376	3.97 ± 0.21	0.025; 0.930	6.87 ± 0.48	0.167; 0.551	7.00 ± 0.49	0.256; 0.357	7.37 ± 0.57	0.234; 0.401

*Note:* n.a.—it does not apply. *p* < 0.05 together with its corresponding r is **bolded**. Pearson correlation coefficients with clean swimming time, and mean cycle velocity is partial correlation coefficients with transition velocity for the respective laps as control variable. Spearman’s rho rank correlation coefficients for mean cycle length and number of strokes on lap 1 with clean swimming time.

**Table 5:** Descriptive statistics (mean ± standard deviation) and partial correlation analysis of 5 m turn in segment time with underlying components.

Segments and Factors	Total	r; *p*-Value	Lap 1	r; *p*-Value	Lap 2	r; *p*-Value	Lap 3	r; *p*-Value
*5 m turn-in time (s)*	9.60 ± 0.62	n.a.	3.03 ± 0.20	n.a.	3.23 ± 0.24	n.a.	3.34 ± 0.22	0.261; 0.368
Mean cycle velocity (m/s)	1.48 ± 0.14	−0.236; 0.417	1.56 ± 0.17	−0.445; 0.111	1.50 ± 0.19	−0.256; 0.377	1.38 ± 0.16	−0.500; 0.068
Mean cycle length (m/cycle)	1.80 ± 0.16	−0.431; 0.109	1.82 ± 0.18	−0.474; 0.074	1.87 ± 0.23	−0.451; 0.092	1.72 ± 0.15	−0.288; 0.298
Mean cycle rate (cycles/min)	49.43 ± 4.87	−0.409; 0.130	51.43 ± 4.74	−0.366; 0.180	48.55 ± 5.88	−0.339; 0.217	48.32 ± 5.19	**−0.521**; **0.046**
Number of cycles	7.29 ± 0.73	0.005; 0.985	2.37 ± 0.30	−0.209; 0.455	2.51 ± 0.44	0.054; 0.849	2.40 ± 0.32	−0.160; 0.568
Glide distance (m)	2.43 ± 0.54	0.396; 0.143	0.83 ± 0.43	0.300; 0.277	0.71 ± 0.39	0.115; 0.683	0.89 ± 0.37	0.222; 0.427

*Note:* n.a.–it does not apply. *p* < 0.05 together with its corresponding r is **bolded**. Pearson correlation coefficients with 5 m turn-in time, and swimming velocity is partial correlation coefficients with clean swimming time of the respective laps as control variable. Spearman’s rho rank correlation coefficients for glide distance total for all turns with 5 m turn-in time.

**Table 6:** Descriptive statistics (mean ± standard deviation) and correlation analysis of the 10 m turn-out segment time with underlying components.

Segments and Factors	Total	r; *p*-Value	Lap 2	r; *p*-Value	Lap 3	r; *p*-Value	Lap 4	r; *p*-Value
*10 m turn-out time (s)*	19.13 ± 0.95	n.a.	6.19 ± 0.31	n.a.	6.36 ± 0.36	n.a.	6.57 ± 0.30	n.a.
Pivot time (s)	2.64 ± 0.22	0.116; 0.680	0.86 ± 0.08	−0.004; 0.989	0.88 ± 0.08	0.315; 0.253	0.90 ± 0.10	0.209; 0.455
Breakout time (s)	17.15 ± 1.75	−0.087; 0.758	5.73 ± 0.61	−0.093; 0.742	5.76 ± 0.60	−0.115; 0.682	5.66 ± 0.60	0.033; 0.907
Breakout distance (m)	27.54 ± 2.62	−0.491; 0.063	9.43 ± 0.92	−0.475; 0.074	9.23 ± 0.94	**−0.525**; **0.044**	8.88 ± 0.81	−0.379; 0.164
Peak velocity (m/s)	2.96 ± 0.14	**−0.733**; **0.002**	3.00 ± 0.15	**−0.587**; **0.022**	2.98 ± 0.17	**−0.725**; **0.002**	2.91 ± 0.13	**−0.688**; **0.005**
Transition velocity (m/s)	1.31 ± 0.07	**−0.709**; **0.003**	1.34 ± 0.10	**−0.698**; **0.004**	1.33 ± 0.06	**−0.535**; **0.040**	1.27 ± 0.07	**−0.755**; **0.001**
Transition cycle length (m/cycle)	1.64 ± 0.12	−0.353; 0.196	1.64 ± 0.14	−0.261; 0.347	1.69 ± 0.14	−0.233; 0.403	1.58 ± 0.12	−0.470; 0.077
Transition cycle rate (cycles/min)	48.36 ± 3.30	−0.166; 0.554	49.00 ± 4.17	−0.344; 0.209	47.61 ± 3.08	−0.089; 0.753	48.47 ± 3.85	−0.011; 0.968

*Note:* n.a.—it does not apply. *p* < 0.05 together with its corresponding r is **bolded**. Pearson correlation coefficients with 10 m turn-out time.

**Table 7:** Descriptive statistics (mean ± standard deviation) and partial correlation analysis of the finish segment time with underlying components.

Segments and Factors	Total	r; *p*-Value
*95–100 m finish time (s)*	3.47 ± 0.23	n.a.
Mean cycle velocity (m/s)	1.30 ± 0.13	−0.121; 0.679
Mean cycle length (m/cycle)	1.57 ± 0.18	−0.319; 0.246
Mean cycle rate (cycles/min)	50.06 ± 6.03	−0.234; 0.400
Glide distance	0.91 ± 0.30	0.069; 0.808
Number of cycles	2.67 ± 0.40	0.191; 0.495

*Note:* n.a.—it does not apply. Pearson correlation coefficients with 95–100 m finish time, and mean cycle velocity is partial correlation coefficient with clean swimming time of the respective lap as control variable.

**Table 8:** Mean swimming velocity (m/s) ± standard deviation for each 5 m segment during the 100 m breaststroke and correlation coefficients with the finishing time.

	0–5 m	r; *p*-Value	5–10 m	r; *p*-Value	10–15 m	r; *p*-Value	15–20 m	r; *p*-Value	20–25 m	r; *p*-Value
Lap 1	3.36 ± 0.16	**−0.879**; **<0.001**	2.18 ± 0.17	**−0.745**; **0.002**	1.40 ± 0.10	**−0.776**; **0.001**	1.49 ± 0.07	**−0.896**; **<0.001**	1.66 ± 0.11	**−0.731**; **0.003**
Lap 2	1.87 ± 0.08	**−0.625**; **0.013**	1.43 ± 0.09	**−0.783**; **0.001**	1.43 ± 0.08	**−0.933**; **<0.001**	1.42 ± 0.07	**−0.730**; **0.003**	1.55 ± 0.12	−0.531; 0.051
Lap 3	1.81 ± 0.09	**−0.651**; **0.009**	1.40 ± 0.10	**−0.730**; **0.003**	1.38 ± 0.06	**−0.978**; **<0.001**	1.37 ± 0.07	**−0.775**; **0.001**	1.50 ± 0.10	−0.138; 0.639
Lap 4	1.78 ± 0.08	−0.346; 0.207	1.34 ± 0.08	**−0.861**; **<0.001**	1.33 ± 0.07	**−0.896**; **<0.001**	1.32 ± 0.06	**−0.777**; **0.001**	1.45 ± 0.10	−0.373; 0.190

Pearson correlation coefficients for 0–5 m on each lap and partial correlation coefficients for the remaining 5 m intervals on each lap. *p* < 0.05 together with its corresponding r is **bolded**.

**Table 9:** Partial contribution (mean ± standard deviation) from the different segments to the finishing time.

Segments and Factors	Total	Lap 1	Lap 2	Lap 3	Lap 4
*Start segment*					
15 m time (%)	11.39 ± 0.22	53.64 ± 0.74	n.a.	n.a.	n.a.
Reaction time (%)	1.05 ± 0.05	4.96 ± 0.28	n.a.	n.a.	n.a.
Flight time (%)	0.59 ± 0.09	2.79 ± 0.43	n.a.	n.a.	n.a.
Underwater time (%)	7.67 ± 0.86	36.10 ± 4.08	n.a.	n.a.	n.a.
Water breakout time (%)	9.31 ± 0.91	43.85 ± 4.36	n.a.	n.a.	n.a.
*Turn segment*					
Total turns (%)	44.30 ± 0.58	n.a.	n.a.	n.a.	n.a.
5 m turn-in (%)	14.80 ± 0.38	21.96 ± 0.45	19.62 ± 0.66	19.66 ± 0.65	n.a.
Pivot time (%)	4.08 ± 0.44	n.a.	5.22 ± 0.61	5.20 ± 0.54	5.11 ± 0.65
Water breakout time (%)	26.53 ± 3.09	n.a.	34.92 ± 3.95	34.03 ± 4.18	32.20 ± 3.79
10 m turn-out (%)	25.43 ± 0.74	n.a.	32.41 ± 0.99	32.28 ± 0.96	32.20 ± 0.92
*Start + turn segment*					
15 m time + total turns (%)	55.70 ± 0.61	n.a.	n.a.	n.a.	n.a.
Water breakout time, start + turns (%)	35.85 ± 3.98	43.85 ± 4.64	37.63 ± 1.01	37.48 ± 1.15	37.31 ± 1.01
*Swim segment*					
Clean swimming (%)	38.93 ± 0.50	24.40 ± 0.58	42.74 ± 0.70	42.86 ± 0.75	42.98 ± 0.47
*Finish segment*					
95–100 m (%)	5.36 ± 0.18	n.a.	n.a.	n.a.	19.71 ± 0.64

Note: n.a.—it does not apply. 10 m out (%) do not include pivot time.

## References

[1] Mason B.R., Formosa D.P., Seifert L., Chollet D. (2010). Competition analysis. World Book of Swimming: From Science to Performance.

[2] Smith D.J., Norris S.R., Hogg J.M. (2002). Performance evaluation of swimmers: Scientific tools. Sports Med..

[3] Morais J.E., Marinho D.A., Arellano R., Barbosa T.M. (2019). Start and turn performances of elite sprinters at the 2016 European Championships in swimming. Sports Biomech..

[4] Tor E., Pease D.L., Ball K.A., Hopkins W.G. (2014). Monitoring the effect of race-analysis parameters on performance in elite swimmers. Int. J. Sports Physiol. Perform..

[5] Thompson K.G., Haljand R., MacLaren D.P. (2000). An analysis of selected kinematic variables in national and elite male and female 100-m and 200-m breaststroke swimmers. J. Sports Sci..

[6] Tor E., Pease D.L., Ball K.A. Characteristics of an elite swimming start. Proceedings of the 12th International Symposium for Biomechanics and Medicine in Swimming.

[7] Vantorre J., Seifert L., Fernandes R.J., Boas J.P.V., Chollet D. (2010). Kinematical profiling of the front crawl start. Int. J. Sports Med..

[8] Peterson Silveira R., Stergiou P., Figueiredo P., Castro F.S., Katz L., Stefanyshyn D.J. (2018). Key determinants of time to 5 m in different ventral swimming start techniques. Eur. J. Sport Sci..

[9] Garcia-Ramos A., Feriche B., de la Fuente B., Arguelles-Cienfuegos J., Strojnik V., Strumbelj B., Stirn I. (2015). Relationship between different push-off variables and start performance in experienced swimmers. Eur. J. Sport Sci..

[10] Veiga S., Roig A. (2016). Underwater and surface strategies of 200 m world level swimmers. J. Sports Sci..

[11] Lyttle A.D., Mason B. (1997). A kinematic and kinetic analysis of the freestyle and butterfly turns. J. Swim. Res..

[12] Veiga S., Roig A., Gomez-Ruano M.A. (2016). Do faster swimmers spend longer underwater than slower swimmers at World Championships?. Eur. J. Sport Sci..

[13] Kennedy P., Brown P., Chengalur S.N., Nelson R.C. (1990). Analysis of male and female olympic swimmers in the 100-meter events. Int. J. Sport Biomech..

[14] Chengalur S.N., Brown P.L. (1992). An analysis of male and female Olympic swimmers in the 200-meter events. Can. J. Sport Sci..

[15] Mason B.R. Biomechanical race analysis. Proceedings of the 31st American Swimming Coaches Association Annual World Clinic.

[16] Wakayoshi K., Nomura T., Takahashi G., Mutoh Y., Miyashita M., MacLaren D., Reilly T., Lees A. (1992). Analysis of swimming races in the 1989 Pan Pacific swimming championships and 1988 Japanese Olympic trials. Biomechanics and Medicine in Swimming: Swimming Science VI.

[17] Robertson E., Pyne D., Hopkins W., Anson J. (2009). Analysis of lap times in international swimming competitions. J. Sports Sci..

[18] Kjendlie P.-L., Haljand R., Fjortoft O., Stallman R.K. (2006). The temporal distribution of race elements in elite swimmers. Port. J. Sport Sci..

[19] Thayer A.L., Hay J.G. (1984). Motivating turn and start improvement. Swim. Tech..

[20] Veiga S., Roig A. (2017). Effect of the starting and turning performances on the subsequent swimming parameters of elite swimmers. Sports Biomech..

[21] Mooney R., Corley G., Godfrey A., Osborough C., Quinlan L., ÓLaighin G. (2015). Application of video-based methods for competitive swimming analysis: A systematic review. Sport Exerc. Med. Open J..

[22] Marinho D.A., Barbosa T.M., Neiva H.P., Silva A.J., Morais J.E. (2020). Comparison of the start, turn and finish performance of elite swimmers in 100 m and 200 m races. J. Sports Sci. Med..

[23] Veiga S., Cala A., Mallo J., Navarro E. (2013). A new procedure for race analysis in swimming based on individual distance measurements. J. Sports Sci..

[24] Haner S., Svärm L., Ask E., Heyden A. Joint under and over water calibration of a swimmer tracking system. Proceedings of the International Conference on Pattern Recognition Applications and Methods—(Volume 2).

[25] Hopkins W.G. A Scale of Magnitudes for Effect Statistics. http://www.sportsci.org/resource/stats/effectmag.html.

[26] Takeda T., Sakai S., Takagi H., Okuno K., Tsubakimoto S. (2017). Contribution of hand and foot force to take-off velocity for the kick-start in competitive swimming. J. Sports Sci..

[27] Taladriz S., de la Fuente-Caynzos B., Arellano R. (2016). Analysis of angular momentum effect on swimming kick-start performance. J. Biomech..

[28] Sakai S., Sekiya K., Takeda T., Takagi H. Kinetic analysis of start motion on starting block in competitiev swimming. Proceedings of the 34 International Conference of Biomechanics in Sport.

[29] Tor E., Pease D.L., Ball K.A. (2015). Key parameters of the swimming start and their relationship to start performance. J. Sports Sci..

[30] Seifert L., Vantorre J., Chollet D. (2007). Biomechanical analysis of the breaststroke start. Int. J. Sports Med..

[31] Naemi R., Easson W.J., Sanders R.H. (2010). Hydrodynamic glide efficiency in swimming. J. Sci. Med. Sport.

[32] Marinho D.A., Barbosa T.M., Rouboa A.I., Silva A.J. (2011). The hydrodynamic study of the swimming gliding: A two-dimensional computational fluid dynamics (CFD) analysis. J. Hum. Kinet..

[33] Vilas-Boas J.P., Costa L., Fernandes R.J., Ribeiro J., Figueiredo P., Marinho D., Silva A.J., Rouboa A., Machado L. (2010). Determination of the drag coefficient during the first and second gliding positions of the breaststroke underwater stroke. J. Appl. Biomech..

[34] Craig A.B., Pendergast D.R. (1979). Relationships of stroke rate, distance per stroke, and velocity in competitive swimming. Med. Sci. Sports.

[35] Craig A.B., Skehan P.L., Pawelczyk J.A., Boomer W.L. (1985). Velocity, stroke rate, and distance per stroke during elite swimming competition. Med. Sci. Sports Exerc..

[36] Hellard P., Dekerle J., Avalos M., Caudal N., Knopp M., Hausswirth C. (2008). Kinematic measures and stroke rate variability in elite female 200-m swimmers in the four swimming techniques: Athens 2004 Olympic semi-finalists and French National 2004 Championship semi-finalists. J. Sports Sci..

